# Governmental Investments in Hospital Infrastructure Among Regions and Its Efficiency in China: An Assessment of Building Construction

**DOI:** 10.3389/fpubh.2021.719839

**Published:** 2021-10-20

**Authors:** Tianjiao Lan, Ting Chen, Yifan Hu, Yili Yang, Jay Pan

**Affiliations:** ^1^HEOA Group, West China School of Public Health and West China Fourth Hospital, Sichuan University, Chengdu, China; ^2^Institute for Healthy Cities and West China Research Center for Rural Health Development, Sichuan University, Chengdu, China

**Keywords:** hospital infrastructure, government investment, equity, efficiency, China

## Abstract

Hospital infrastructure has been addressed as the prerequisite of healthcare delivery which intensively affects medical quality. Over the past decade, China has proposed a series of investment plans for hospital infrastructure in order to promote healthcare development in underdeveloped regions. Focusing on the construction of hospital buildings as the key component of hospital infrastructure, this study aims to examine whether the investment efficiency is lower where a government prioritizes equity and to explore what kind of geographical predispositions should be embedded in governmental investment plans for hospital infrastructures from the perspectives of both investment equity and efficiency. Relevant data from 330 governmental-invested hospital building construction projects in Sichuan province, China, from 2009 to 2018 were collected. Concentration index was used to evaluate the equity in the distribution of the investments. Tobit model was employed to explore the relationship between regional economic development and investment efficiency measured by an integrated approach of principal component analysis and data envelopment analysis. The results demonstrated a slight concentration of governmental investments in economically developed regions, while a negative association with regional economic development was identified with investment efficiency. Our study illustrated the investment efficiency was higher where a government prioritized equity and provided empirical evidences on switching governmental investment predisposition in the aspect of healthcare infrastructure construction toward less developed regions in China from the perspectives of both investment allocation equity and efficiency, which would further assist in the formulation of region-specific policies and strategies for underdeveloped regions.

## Introduction

Healthcare facility infrastructure, including physical, technical, and organizational components or assets, has been emphasized as the prerequisite for the delivery of healthcare services (1). The deficiency of physical infrastructure, such as buildings, beds, medical equipment, logistics equipment or other fixed assets, could pose a huge obstacle for healthcare facilities to expand their medical services as well as improving healthcare quality (2, 3). However, the distribution of healthcare facility infrastructure typically demonstrated large discrepancies among different regions especially within developing countries which were often constrained by limited investments in healthcare sectors (4), thus ultimately leading to inequities in the distribution of medical facilities (5–7).

Governmental investment, as a method of resource reallocation, has been highlighted as an effective way to reduce discrepancies among different regions thus improving social equity (8). The governmental investment in healthcare facilities infrastructure in less developed regions has been believed to be an essential strategy for minimizing healthcare inequity (9). In addition to equity issues, efficiency should also be taken into consideration throughout the decision-making procedures of governmental investments (10). In the healthcare sector, investment efficiency has been defined as the production of the maximum health gains as the result of a given amount of healthcare input (11).

However, through the ages, multiple countries have simply insisted that the governmental investment in health facilities infrastructure should be more focused on less developed regions from the perspective of equity (12–14). The absence of investment efficiency in investment decision procedure makes us consider that is efficiency lower where a government prioritizes equity? The generally accepted assumption of the production function has indicated that an increased input would result in a reduced marginal output (15). Thus, based on such assumption, it could be theoretically predicted that both higher investment efficiency and better social equity would be probably achieved via predisposed resource allocation toward underdeveloped areas. However, the evidence on this assumption from health care is far from clear, not to mention the conduction of relevant evaluations on governmental investments in terms of healthcare facilities infrastructure among various regions from the perspectives of both investment equity and efficiency.

Therefore, to bridge the gap in existing literatures, this study was designed to answer the following two research questions. First, to examine whether the investment efficiency is lower where a government prioritizes equity. Second, to explore what kind of geographical predispositions should be embedded in governmental investment plans for hospital infrastructures in China from the perspectives of both investment equity and efficiency. Relevant data on 330 governmental-invested hospital building construction projects in Sichuan province, China, from 2009 to 2018, were collected. We firstly used the concentration index to describe the current distribution equity of governmental investments. Then the tobit regression was adopted to address the first research question by exploring the relationship between regional economic development and investment efficiency which was measured by PCA-DEA model. Finally, we combined the two analysis results to shed light on what kind of geographical predispositions should be embedded in governmental investment plans for hospital infrastructures in China from the perspectives of both investment equity and efficiency, i.e., the second research question.

Rather than the whole hospital physical infrastructure, our study specifically focused on the construction of healthcare facilities. Hospital building construction was selected as our study focus for two reasons. First, particular attention should be paid on healthcare facility construction which serves as the fundamental component of hospital infrastructure as well as the prerequisite for all the other components, Second, it should be noted that hospital building construction is the most essential phase reflective of the actual implementation of governmental investment plans for hospital infrastructure promotion in China. Previous studies have investigated the operational status of healthcare facilities via evaluating multiple more specific aspects related to healthcare facility infrastructure such as communication devices, waste disposal and water supplier devices (1, 5–7). However, as in China all these types of devices would be generally purchased as part of hospitals' own finance budgets without receiving any compensation from governmental investments, thus, we merely adopted hospital construction status in this study as the key indicator for obtaining a more comprehensive understanding about governmental investments in the aspect of healthcare infrastructure.

This study was expected to contribute to the relevant literature and health policy planning. First, focusing on the hospital infrastructure, this is, to our best knowledge, the first study to examine whether the investment efficiency is lower where a government prioritizes equity. Second, the development of healthcare infrastructure had been tightly constrained by limited governmental budgets, especially for developing countries like China. Our study was expected to provide evidence-based implications of what kind of geographical predispositions should be embedded in governmental investment plans for hospital infrastructures in China from the perspectives of both investment equity and efficiency. Findings from such study would facilitate the development of hospital infrastructure in the context of limited governmental budgets.

An overview of governmental investments in hospital building construction in China was briefly described in Text A.1 (Supplementary Material).

## Literature Review

### Efficiency and Equity

The balance between equity and efficiency remains a concern among health economists (16, 17). Despite of Adam Smith's belief that competitive markets will make society more equitable and efficient, health care does not often satisfy the requirements for competitive markets (18). Thus, whether equity and efficiency of governmental investment in health care can be achieved simultaneously need empirical studies.

While growing studies have focused on the equity and efficiency of health system (19–22), however, very limited studies have sought to evaluate the governmental investment in health care from the perspective of both equity and efficiency. Liu and He (23) assessed the equity and efficiency of financing for total health expenditure in China; Sun and Luo (24) evaluated the equity and efficiency of health resources allocation in China; Li et al. (25) evaluated the equity and efficiency of healthcare resource allocation of Chinese medicine in mainland China. Caroline et al. (26) proposed the priority setting of health interventions in Ghana based on a novel equity-efficiency tradeoff framework. However, all these previous studies failed to the answer the question: whether the efficiency is lower where a government prioritizes equity?

As for healthcare infrastructure in China, at present, no previous study has evaluated the equity and efficiency of governmental investment in hospital infrastructure. Most studies mainly assessed the locations of existing healthcare facilities from the perspective of distribution equity, based on which consensus has been reached that strengthening healthcare investment in less developed regions at governmental level should be addressed as the key strategy to achieving enhanced distribution equity among different regions (27–33).

### Efficiency Measure

Hollingsworth reviewed 188 published papers on efficiency measurement in health care and concluded that data envelopment analysis (DEA) and stochastic frontier analysis (SFA) are mostly used methods to measure efficiency in health area (34). DEA and SFA belong to a class of methodologies for measuring efficiency called “frontier analysis” which compares a firm's (e.g., hospital) use of actual inputs and outputs to efficient combinations of multiple inputs and/or outputs (35). The two methods use different approaches to calculating the “frontier” of efficient combinations used for comparison. Compared with SFA, a parametric model, the main advantage of DEA remains its non-parametric estimation, which relax the assumption required by unbiased efficiency estimation [see chapter 2 of (36) for technical details].

In practice, the selection of inputs and outputs in DEA or SFA depends on the research purpose. Victor and Kim reviewed 57 studies using DEA in health care (37). They found that input indicators were usually selected from capacity dimension (e.g., number of beds), labor dimension (number of physicians), and expenses dimension (e.g., total supplies cost); output indicators were usually selected from activity dimension (e.g., number of inpatient and outpatient visits) and quality dimension (e.g., mortality rate).

## Materials and Methods

### Framework

The framework of our study has been developed based on the logic models proposed by previous studies (38, 39), which was adopted for evaluating hospital construction projects as the predominant aspect of hospital infrastructure. The logic model has been widely used to visually depict the hypothesized relationships among project resources, project activities, and the results the project are expected to achieve (40, 41). In the healthcare sector, Donabedian (42) proposed a logic model as a potent tool for evaluating the quality of healthcare which contained the structures, process and outcomes of healthcare delivery. The terminologies adopted in our study were obtained from his model as our study has the similarity with the previous study in terms of evaluating the productive procedures of healthcare delivery.

**Figure 1:**
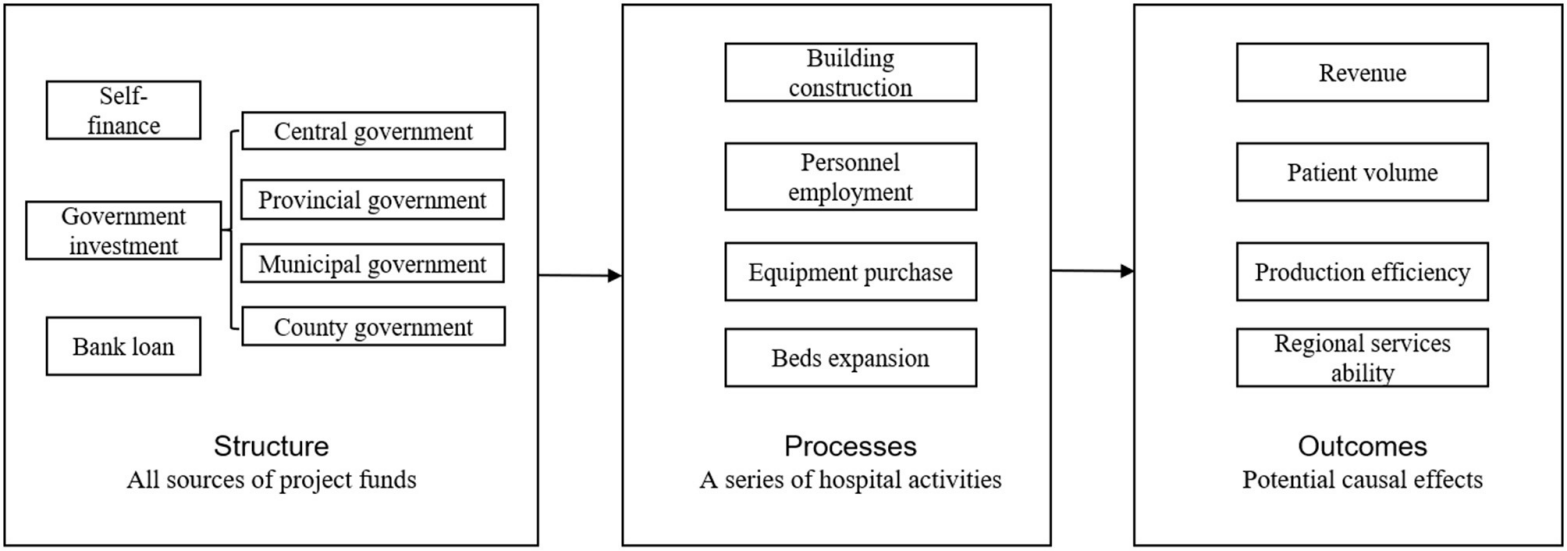
Study framework.

As shown in Figure 1, we divided the hospital building construction project into three stages, namely structure, processes and outcomes. Structure as the initial phase denotes all the resources required for the project, thus including all the funding resources obtained. The second phase contains a series of hospital activities after receiving project funds, thus were called processes. The hospital activities were summarized as four aspects including building construction, personnel employment, equipment purchase, and hospital beds expansion. Combined with the results of expert consultation and relevant literatures (37, 43–46), five variables were selected as indicators reflective of these four aspects, as shown in Table 1.

**Table 1:** Definition of input and output variables.

**Variable**	**Definition**
**Processes (Inputs in DEA)**	
Planned building area	Building area planned according to the investment plan
Total number of hospital beds	Actual functional status of beds in a hospital
Total value of equipment above 10,000 Yuan	Summing up the values of any equipment whose value is above 10,000 Yuan, including medical equipment and hospital logistics equipment
Number of healthcare workers	Including physicians, pharmacists, nurses, and medical technicians
Proportion of staff with senior professional titles	Proportion of staff with senior professional titles among all staffs in a hospital
**Outcomes (Outputs in DEA)**	
Outpatient revenue per 10,000 population	Annual outpatient revenue dividing by the population of the county in which the hospital is located
Inpatient revenue per 10,000 population	Annual inpatient revenue dividing by the population of the county in which the hospital is located
Outpatient and emergency department patient volume per 10,000 population	Annual outpatient and emergency department patient volume dividing by the population of the county in which the hospital is located
Number of inpatient discharges per 10,000 population	Number of annual inpatient discharges dividing by the population of the county in which the hospital is located
Number of inpatient surgical procedures per 10,000 population	Number of annual inpatient surgical procedures dividing by the population of the county in which the hospital is located
Bed days per physician per day	Total bed days per day for a hospital divided by the number of physicians
Bed turnover rate	Average number of patients cared for a bed during 1 year
Office visits per physician per day	A doctor's visit per day
Admission ratio within municipality	A ratio of actual frequency of admissions (Total frequency of admissions within a municipality) to theoretical frequency of admissions. The theoretical frequency of admissions is calculated as follows: $\begin{array}{l} \frac{\text{Frequency of admissions within the province}}{\text{Population of the province}}\\ \text{*}\;\text{Population of a municipality} \end{array}$

As for the outcomes part, we assumed that the hospital activities as mentioned above would lead to outcomes in four aspects, namely revenue, patient amount, productive efficiency, and regional service capacity. On one hand, the increased amount of hospital beds (47), personnel (48), and equipment (49) would improve the revenues and patient volumes into hospitals. In addition, the expansion of hospitals could increase their revenues and patient volumes by gaining a greater competitive advantage in the hospital market (50). On the other hand, according to the SCP paradigm drawn from industrial organization theory (51), changes in hospital market structure would lead to changes in market performance. Specifically, governmental investment in hospital building construction, as a policy shock to the hospital market, would change the structure of the market and could eventually increase hospital production efficiency (52) as well as regional service capacity (53) within the market through hospital competition. Following a review listing the typical outputs selected in the previous studies (37), we chose outpatient and inpatient revenue to reflect hospital revenue; we selected outpatient and emergency department patient volume, number of inpatient discharges, and number of inpatient surgical procedures to represent patient volume. After consultation with experts in Health Commission of Sichuan Province, we employed bed days per physician per day, bed turnover rate, and office visits per physician per day to reflect production efficiency; and we used admission ratio within municipality to represent regional services ability. Therefore, nine variables were selected as indicators reflective of four aspects of the outcomes, as shown in Table 1^1^.

We used concentration index to describe the distribution equity of the governmental investment. Despite that governmental investment in hospital building construction projects came from four governmental sources, we only included central and provincial governmental investments as the main resources based on two considerations. First, the data quality of municipal and county investment was too poor to be adopted for analysis^2^. Second, the amount of the investment from municipal or county governments between different regions was not comparable^3^. Based on the processes and outcomes parts in our framework, we further used data envelopment analysis (DEA) along with principal component analysis (PCA) to compute the project efficiency. The project efficiency is very similar with the governmental investment efficiency in our study, and was defined as the maximum population health benefit after the completion of the hospital building construction project given the condition of certain inputs (processes variables in the framework) of supporting health resources. Thus, to avoid confusion, we employed investment efficiency to refer to project efficiency throughout the paper. The tobit regression was then adopted to address the first research question by exploring the relationship between regional economic development and investment efficiency which was measured by PCA-DEA model. Finally, we combined the two analysis results to shed light on what kind of geographical predispositions should be embedded in governmental investment plans for hospital infrastructures from the perspectives of both investment equity and efficiency, i.e., the second research question.

### Study Area

**Figure 2:**
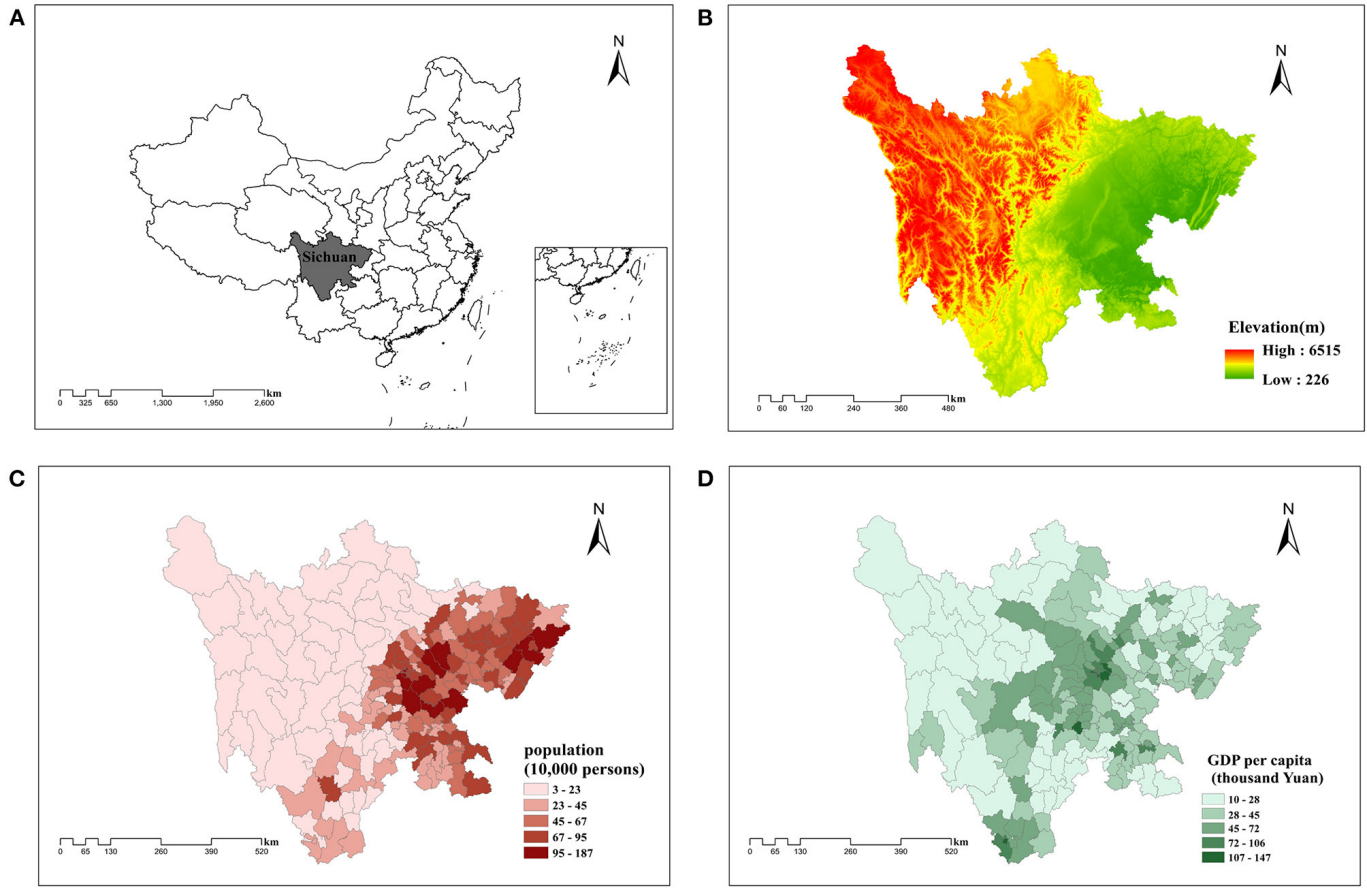
**(A)** Geographic position, **(B)** topography, **(C)** demography, and **(D)** economic development in Sichuan Province, China. The data for geographic position and topography are from AutoNavi. Copyright 2021 by Amap.com. The data for demography and economic development are from The Statistical Yearbook of Sichuan Province, by Sichuan Provincial Bureau of Statistics, 2019 (http://tjj.sc.gov.cn/tjnj/cs/2019/zk/indexch.htm). Copyright 2021 by China Statistics Press.

Using Sichuan Province as the study area, this study was designed to reflect the overall situation of healthcare infrastructure construction in China to a certain extent. Sichuan Province is a southwestern province in China (Figure 2A), where the land area and GDP per capita ranked fifth and nineteen, respectively, among 31 provinces of Mainland China, with a population of 83.41 million reported in 2018 (55). As indicated by Figures 2B–D, eastern Sichuan is characterized by plains, dense population, and high-level economic development, while western Sichuan is in the opposite situation (49). Such geographical characteristics and economic development status in Sichuan province made this region an ideal study area for simulating the nationwide situation. Similarly with Sichuan Province, approximately 41% of the entire population in China reside in the eastern China which has experienced rapid economic development with its topography characterized by plains and hills. In contrast, the western China has a much lower economic development pace which is sparsely populated and covered by mountains and plateaus.

### Study Period

As the construction of hospital buildings is a time-consuming process, a time slot was set between the initiation of project and the production of related activities and outcomes in order to evaluate the overall investment efficiency. Based on the consultation with Health Commission of Sichuan Province, we finally set 2 years as the time slot^4^. As the result, investment data from 2009 to 2016 was adopted for assessing the investment equity while other relevant data was collected from 2009 to 2018 for evaluating the overall investment efficiency from 2009 to 2016.

### Data Sources

The Department of Statistics of Health Commission of Sichuan Province (http://wsjkw.sc.gov.cn/scwsjkw/zsdw1/2019/3/22/9eb15f774d5549ff9a99c979fa8a0e2b.shtml) provided the information about all governmental-invested hospital building construction projects which was retrieved from its information management system. The data from 2009 to 2016 contained the names and locations of governmental-invested hospitals, the planned building areas, as well as the amount of investment from all funding sources.

Relevant hospital-level data from 2009 to 2018 were administrative data extracted from the hospital annual report and also provided by Health Commission of Sichuan Province, which included each governmental-invested hospital's basic and delivery information. The hospital's basic information included the total number of hospital beds, total value of equipment above 10,000 Yuan, number of healthcare workers, hospital level (primary, secondary, tertiary, and un-graded), whether general, hospital building area, and proportion of staff with senior professional titles. The hospital's delivery information included outpatient revenue, inpatient revenue, outpatient and emergency department patient volume, number of inpatient discharges, number of inpatient surgical procedures, bed days per physician per day, bed turnover rate, and office visits per physician per day.

The county-level data from 2009 to 2018 were extracted from the statistical yearbook of Sichuan Province, including each county's information about its population, urbanization rate, and GDP per capita.

We used the unique hospital code and county code to match the hospital-level data and county-level data to governmental-invested hospital building construction projects, separately. Our sample contained 330 governmental-invested hospital building construction projects which was further adopted for the concentration index computation, among which three projects related to new hospital building construction projects were excluded due to its huge heterogeneity among all kinds of hospital construction projects. Nine projects with missing values were also excluded. After the exclusion step, 318 projects were finally used to explore the relationship between economic development and investment efficiency. All kinds of currencies were adjusted for inflation rates, and measured in 2016 RMB.

### Empirical Strategy

#### Evaluating the Distribution of Government Investment

We used the concentration index (CI) to describe equity in the geographic distribution of government investment in hospital building construction. All of 330 projects from 2009 to 2016 were included in this analysis.

The concentration index, which has been widely used to describe equity (56–58), evaluates the distribution of health resources against economic status (59). The concentration index is defined as twice the area between the concentration curve (cumulative proportion of health resources projected onto the corresponding cumulative proportion of wealth) and diagonal, ranging from −1 to +1. The index value equaling zero implies no socioeconomic inequality, a positive value indicates a concentration of health resources in high economic development regions while a negative value represents a concentration of health resources in low economic development regions.

The amount of investment from government used for CI calculation included both central and provincial governments investment. We pooled all the amount in a county in 1 year, and then divided by the county's population, based on which we calculated the concentration index.

#### Exploring the Relationship Between Regional Economic Development and Efficiency

We first conducted data envelopment analysis along with principal component analysis (PCA-DEA model) in order to compute the investment efficiency. Then, we used tobit model to explore the relationship between regional economic development and investment efficiency.

Data envelopment analysis (DEA) is a non-parametric method for efficiency measure (34). DEA allows for simultaneous consideration of multiple inputs and outputs, which is suitable for measuring the efficiency of complex systems as required in our study. We used BCC model (a kind of DEA model) proposed by Charnes et al. (60) to estimate the investment efficiency. The BCC model is defined as follows:


(1) $$\begin{array}{c} \text{Max}\theta_{\text{k}=}\sum_{r=1}^{s}u_{rk}y_{rk}\\ \sum_{i=1}^{m}v_{ik}x_{ik}=1\\ S.T.\{\begin{array}{c} \sum_{r=1}^{s}u_{rk}y_{rj}-\sum_{i=1}^{m}v_{ik}x_{ij}\leq 0;j=1,\ldots ,n\\ u_{rk}\geq 0,r=1,\ldots ,s\\ v_{ik}\geq 0,r=1,\ldots ,m \end{array} \end{array}$$

where θ_*k*_ is the efficiency of building construction project of hospital *k*. *u*_*rk*_*, v*_*ik*_ represents the coefficients of *r*th output and *i*th input, respectively. The estimated efficiency ranges from 0 to 1, with higher values indicating higher efficiency.

The variables in the processes and outcomes parts in the framework were set as inputs and outputs in the BCC model, respectively. Apart from the planned building area indicator, the values of inputs and outputs were in forms of the difference between the value in the project beginning year and that of 2 years later. Thus, we collected relevant data from 2009 to 2018 to estimate the efficiency of the projects from 2009 to 2016. Despite the uniqueness embedded in our study, most of the inputs and outputs are consistent with literatures (37, 43–46).

Due to the excessive number of outputs in our study, we used principal component analysis (PCA) to reduce the number of variables in advance. In the integrated approach of PCA and DEA (PCA-DEA model), principal component analysis (PCA) was applied to replace the original *s* outputs with a smaller group of principal components. Using principal components instead of the original data does not affect the properties of the DEA model (61). Following Kaiser and Morrison (62, 63), the components selection criteria were described as follows. First, eigenvalue of the component should be more than 1. Second, selected components should account for 80 percent of total variance.

Finally, tobit regression model was applied to explore the relationship between regional economic development and the investment efficiency. Censored efficiency scores (0–1) is not suitable for OLS method, so it is preferable to regress using tobit model. Following Cheng and Zere (45, 64), we transformed the technical efficiency scores into inefficiency scores for convenient computation. After transformation, the censoring point in tobit model was at zero. This transformation of the dependent variable also reversed the signs of the coefficient in the regression. The transformation formula is as follows:


(2) $$\begin{array}{l} Inefficiency\;score=\left(\frac{1}{Technical\;efficiency\;score}\right)-1 \end{array}$$

The tobit model was set as follows:


(3) $$\begin{array}{l} Inefficiency_{i,t}=\beta \text{0+}\beta L\text{og}\left(GDP\;per\;capita_{i,t}\right)\text{+}\gamma^{\prime }H_{i,t}\\ \;+\zeta^{\prime }County_{i,t}\text{+}\varepsilon_{i,t} \end{array}$$

*H*_*i,t*_ is a vector of hospital basic characteristics, including hospital level (primary, secondary, tertiary, and un-graded), whether general, hospital building area, amount of investment from provincial and central government, total number of hospital beds, total value of equipment above 10,000 Yuan, number of healthcare workers, and proportion of staff with senior professional titles. *County*_*i,t*_ is a vector of variables related to county's characteristics, including population and urbanization rate. ε_*i,t*_ is the error term. Given that the original condition of hospitals such as level, size, et al. should be controlled for in the regression model, the *H*_*i,t*_ and *County*_*i,t*_ variables are included with the values in the project beginning year. The robust standard errors were used to correct heteroskedasticity (65).

β is the coefficients of interest. A positive value means that GDP per capita is negatively associated with investment efficiency.

It is worth noting that for continuous variables, we employed the median to describe the data. For normally distributed data, median is consistent with mean (66); for skewed data median is the better summary measure (67). Thus, win-win for median. In this study, all analyses are conducted using R 3.6.3, SPSS 23.0, and DEAP 2.1. *P* < 0.05 is used to determine statistical significance.

## Results

### Equity in the Distribution of Government Investment in Hospital Building Construction

**Figure 3:**
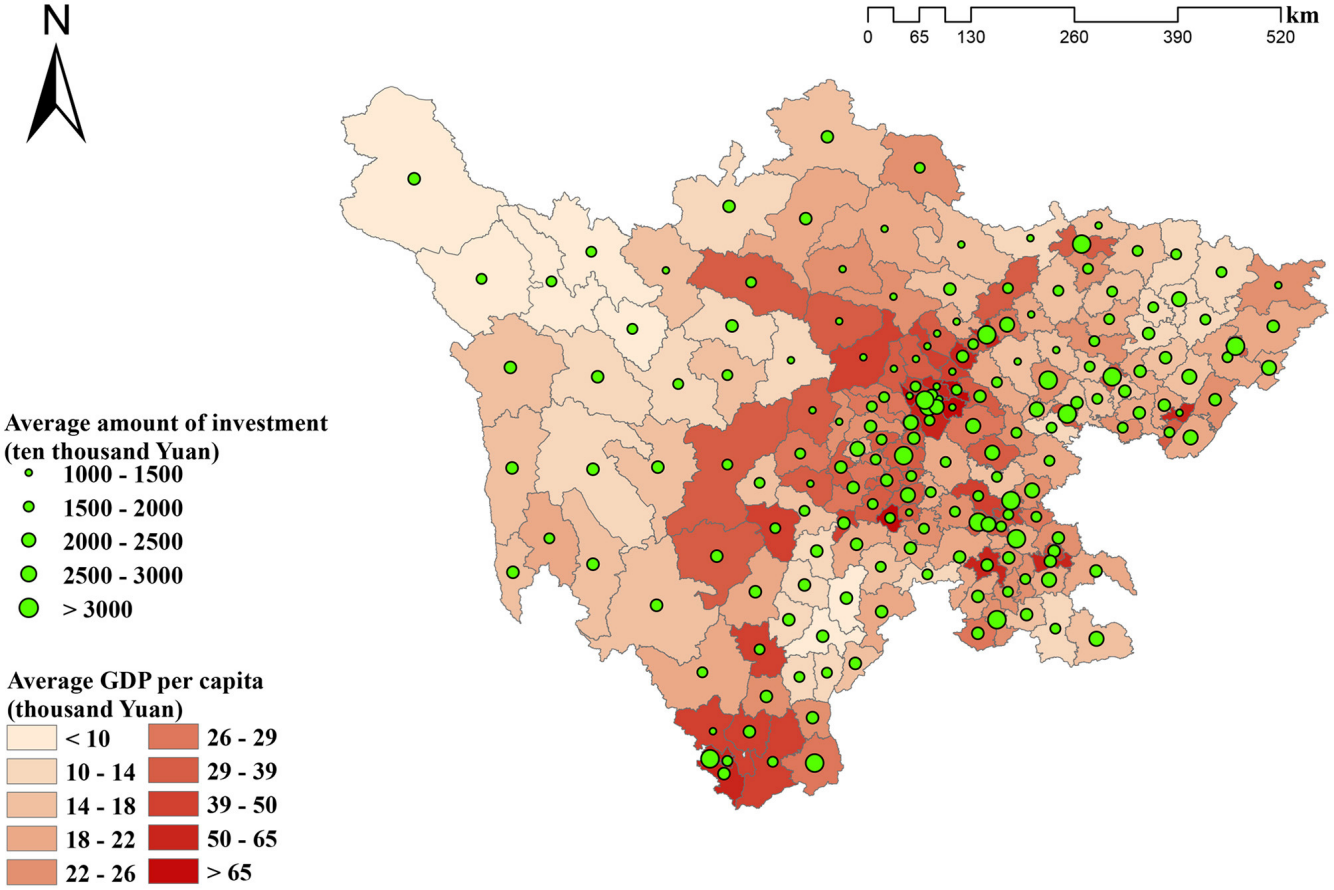
The distribution of average amount of provincial and central governments investment among counties from 2009 to 2016.

Figure 3 depicts the distribution of averaged amount of provincial and central governmental investments among counties from 2009 to 2016. In terms of governmental investments on hospital building construction projects, significant discrepancies were found among different regions, showing that regions with higher averaged amounts of the investment per county mainly clustered in the economically developed eastern region. An opposite situation was identified in the western region. Figure 3 qualitatively implies that the governmental investment varied among regions with different levels of economic development, which ultimately led to investment inequity.

**Figure 4:**
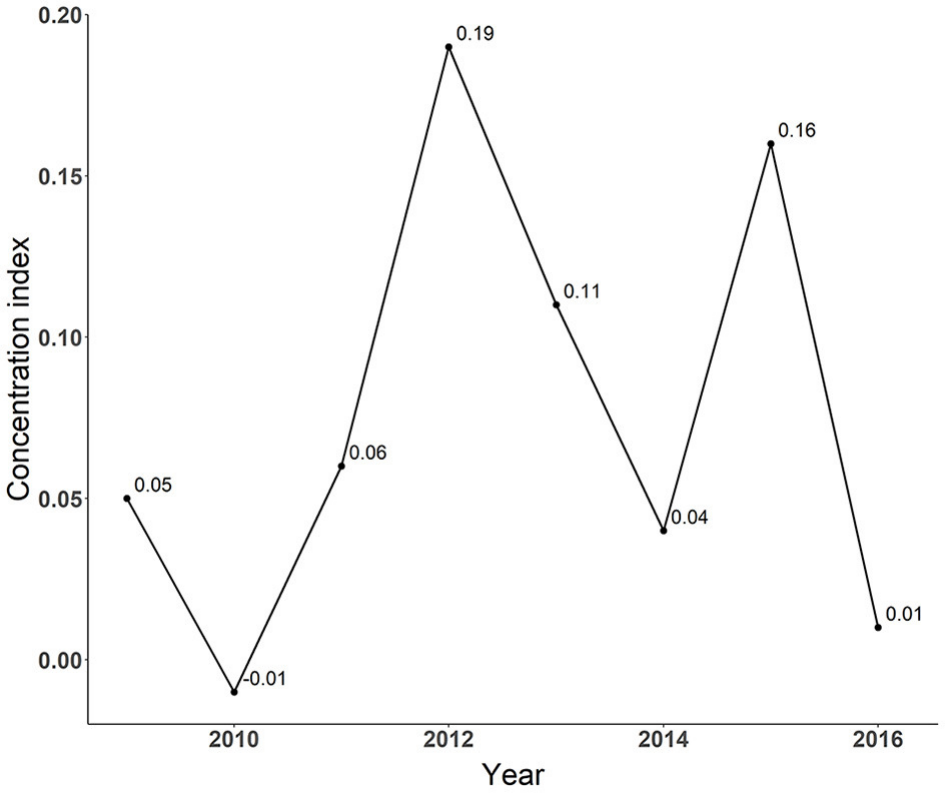
Concentration index of government investment in hospital building construction.

Concentration index was further employed to quantitatively. assess the degree of inequity. Figure 4 reports the results of concentration index. The values ranged from −0.01 to 0.19, all of which were positive except for the negative value in 2010. As a positive concentration index value indicates a concentration of health resources in high economic development regions, the results demonstrated a slight concentration of governmental investment in regions with highly developed economic status, with more than half of its positive values <0.1 (ranging from 0.01 to 0.06).

### The Relationship Between Regional Economic Development and Efficiency of Hospital Building Construction Project

Table 2 shows the descriptive statistics of inputs and outputs in DEA model. The medians of planned building area demonstrated an inverted U-shaped from 2009 to 2016, indicating that the scale of hospital building construction has regained attention in recent years. Apart from bed days per physician per day and admission ratio within municipality, the values of inputs and outputs were found to be significantly positive in most years. The median of total number of hospital beds, for example, ranged from 31 to 70 across years.

**Table 2:** Descriptive statistics of inputs and outputs.

**Variables**	**2009**	**2010**	**2011**	**2012**	**2013**	**2014**	**2015**	**2016**
**Inputs (Processes in framework)**								
Planned building area	12,787 (5,250)	10,545 (6716.25)	9,100 (4139.50)	9,500 (12,720)	9,000 (5,717)	8,710 (14,228)	12,000 (16,050)	13,500 (7,559)
Total number of hospital beds	49.50 (86.25)	40 (94.75)	53 (93.50)	48 (192)	52 (117.50)	31 (73)	70 (184)	32.5 (78.50)
Total value of equipment above 10,000 Yuan	340 (642.25)	327 (719)	175 (998.50)	2,765 (4,980)	764 (3,040)	1697.50 (4247.25)	1,015 (3,035)	121.50 (753.25)
Number of healthcare workers	42.50 (67)	21 (60.50)	34 (71)	83 (157)	74 (78)	79 (143)	51 (125)	38 (55.75)
Proportion of staff with senior professional titles	−24.25 (54.39)	−0.38 (81.85)	−78.70 (48.42)	19.89 (44.07)	55.86 (75.23)	41.24 (120.68)	41.40 (92.85)	56.94 (95.42)
**Outputs (Outcomes in framework)**								
Outpatient revenue per 10,000 population	87.61 (93.84)	136.86 (148.04)	91.73 (326.58)	547.01 (875.30)	189.32 (352.16)	294.74 (366.61)	247.78 (599.78)	151.87 (233.87)
Inpatient revenue per 10,000 population	235.24 (229.84)	322.51 (363.84)	428.79 (771.95)	966.45 (1698.37)	360.28 (662.17)	619.84 (847.08)	733.89 (1032.80)	329.04 (342.30)
Outpatient and emergency department patient volume per 10,000 population	214.60 (478.89)	514.65 (821.24)	466.17 (1383.71)	910.70 (1732.88)	725.16 (1101.92)	1053.72 (1398.80)	546.20 (1365.15)	667.70 (987.46)
Number of inpatient discharges per 10,000 population	29.27 (54.11)	50.48 (64.81)	49.09 (114.00)	74.40 (122.94)	28.28 (51.87)	50.66 (78.67)	53.03 (88.35)	32.64 (53.49)
Number of inpatient surgical procedures per 10,000 population	4.10 (11.45)	5.45 (13.36)	1.90 (20.96)	25.41 (52.19)	10.34 (22.43)	15.73 (25.96)	19.41 (35.66)	5.46 (25.68)
Bed days per physician per day	−0.09 (0.43)	−0.20 (0.46)	−0.42 (0.77)	−0.37 (0.56)	−0.42 (0.51)	−0.40 (0.51)	−0.35 (0.45)	−0.51 (0.37)
Bed turnover rate	−2.72 (14.04)	−0.01 (11.12)	1.01 (4.77)	1.36 (4.85)	−0.20 (8.12)	0.96 (7.10)	0.87 (6.47)	0.46 (7.56)
Office visits per physician per day	0.00 (0.96)	0.56 (1.41)	0.19 (0.85)	0.10 (1.03)	−0.23 (1.32)	−0.13 (0.92)	−0.03 (1.17)	0.03 (1.12)
Admission ratio within municipality	0.81 (12.23)	−1.74 (12.96)	−0.31 (4.68)	−0.74 (5.91)	−1.02 (8.98)	−3.43 (8.06)	1.67 (6.88)	−2.55 (3.77)
*N*	56	56	51	33	27	30	37	28

*Median (interquartile range) is shown in the table*.

*The units of equipment value and revenue is 10,000 Yuan and 1,000 Yuan respectively; the unit of planned building area is square meter*.

*Except planned building area, the values of inputs and outputs are in forms of the difference between the value of project beginning year and that of 2 years later*.

Prior to the adoption of DEA, we conducted principal component analysis (PCA) to reduce the number of outputs. KMO test and Bartlett Spherical Detection were firstly used to detect whether the outputs listed in Table 2 were suitable for PCA. The statistics of KMO test was 0.952 and the *P* value of Bartlett Spherical Detection was <0.001 (Supplementary Table A1), which validated the rationale of Principal Component Analysis for these variables (68, 69). Table 3 shows the principal components from PCA. Five principal components with eigenvalues >1 were selected, which accounted for 82.6% of the total variance. Principal component scores instead of original outputs were used in DEA model.

**Table 3:** Principal components from principal component analysis.

**Principal components**	**Eigenvalues**	**Output variables**
PC 1	3.20	Outpatient revenue per 10,000 population, Inpatient revenue per 10,000 population, Outpatient and emergency department patient volume per 10,000 population
PC 2	2.56	Number of inpatient discharges per 10,000 population, Number of inpatient surgical procedures per 10,000 population
PC 3	2.07	Bed days per physician per day, Office visits per physician per day
PC 4	1.55	Bed turnover rate
PC 5	1.07	Admission ratio within municipality

Following DEA, we employed tobit model to explore the relationship between regional economic development and investment efficiency. Table 4 demonstrates the descriptive statistics of regression variables. Of note, we reported the efficiency score in Table 4 but inefficiency score was employed as outcome variable in tobit regression. The medians of efficiency in most years reached up to 1 (censored point), implying the necessity of tobit model. The median of the GDP per capita ranged from 14,756 Yuan to 27,490 Yuan during 2009 to 2016, with the interquartile ranging from 7,301 to 13,818. The majority (67.9%) of the governmental-invested hospitals were found to be secondary hospitals, followed by primary (25.5%), un-grade (4.1%) and tertiary (2.5%) hospitals. Among them, the general hospitals accounted for 63.5% of the analyzed sample. The medians of the amount of investment from provincial and central governments demonstrated an inversed U-shaped trend ranging from 2,437 ten thousand Yuan to 3,920 ten thousand Yuan, which implied that the scale of governmental investment in single hospital building construction projects regained increased attention. The descriptive statistics of other independent variables are not reported in the text due to the space limitation but can be found in Table 4.

**Table 4:** Descriptive statistics of regression variables.

**Variables**	**2009**	**2010**	**2011**	**2012**	**2013**	**2014**	**2015**	**2016**
**Dependent variable**								
Efficiency scores	1 (0.04)	0.99 (0.07)	0.97 (0.09)	1 (0.01)	1 (0.00)	1 (0.00)	1 (0.05)	0.99 (0.05)
**Explanatory variables**								
GDP per capita	14755.56 (7300.77)	17169.75 (13939.49)	25584.43 (18856.14)	35460.51 (29622.80)	29472.73 (17990.80)	37447.84 (19896.80)	32102.58 (19120.52)	27,490 (13817.75)
Population	5.80 (5.57)	3.46 (5.99)	5.94 (5.60)	6.54 (5.09)	5.95 (3.39)	6.21 (2.98)	6.07 (4.09)	4.57 (4.68)
Urbanization rate	16.32 (7.70)	16.48 (19.02)	25.74 (28.59)	42.86 (40.48)	28.13 (34.87)	41.16 (25.36)	41.76 (31.81)	37.99 (14.02)
**Hospital level *n* (%)**								
Primary	1 (1.79)	2 (3.57)	4 (7.84)	1 (3.03)	0 (0.00)	0 (0.00)	0 (0.00)	0 (0.00)
Secondary	54 (96.43)	49 (87.50)	28 (54.90)	11 (33.33)	17 (62.96)	13 (43.33)	22 (59.46)	22 (78.57)
Tertiary	0 (0.00)	1 (1.79)	12 (23.53)	20 (60.61)	10 (37.04)	17 (56.67)	15 (40.54)	6 (21.43)
Un-grade	1 (1.79)	4 (7.14)	7 (13.73)	1 (3.03)	0 (0.00)	0 (0.00)	0 (0.00)	0 (0.00)
**Whether general *n* (%)**								
No	12 (21.43)	14 (25.00)	27 (52.94)	9 (27.27)	9 (33.33)	11 (36.67)	20 (54.05)	14 (50.00)
Yes	44 (78.57)	42 (75.00)	24 (47.06)	24 (72.73)	18 (66.67)	19 (63.33)	17 (45.95)	14 (50.00)
Hospital building area	16,138 (18070.50)	10031.50 (16559.75)	12,695 (19701)	58,687 (62670)	28,697 (50563.50)	33402.50 (76,599)	30,509 (53,776)	14718.50 (14063.50)
Amount of investment from provincial and central governments	2436.60 (60.16)	2506.79 (174.89)	2103.80 (752.94)	2160.51 (1674.40)	1996.58 (262.71)	2068.57 (2068.57)	1992.15 (962.96)	3,920 (1197.50)
Total number of hospital beds	216 (151.25)	130 (237.75)	310 (358.50)	830 (733)	400 (816.50)	600 (1002.50)	430 (370)	236 (442.50)
Total value of equipment above 10000 Yuan	1188.50 (1366.50)	669 (1,302)	780 (2960.50)	6,014 (13,684)	4,935 (10,390)	6794.50 (16,203.25)	3,760 (8,827)	2172.50 (3,497)
Number of healthcare workers	217.50 (144)	139 (224.75)	148 (304)	770 (739)	418 (778)	624.5 (1034.50)	402 (510)	255 (345.25)
Proportion of staff with senior professional titles	7.67 (3.07)	7.83 (3.46)	7.67 (1.98)	6.81 (2.16)	6.86 (2.29)	7.09 (2.43)	7.20 (1.68)	6.88 (1.78)
*N*	56	56	51	33	27	30	37	28

*n (%): number (percentage)*.

*Unless otherwise indicated, data are expressed as median (interquartile range) in the table*.

*The unit of population is 100,000; the unit of building area is square meter; the units of equipment value and amount of investment from provincial and central governments are 10,000 Yuan*.

*The values of independent variables are captured in the year of project beginning*.

Table 5 shows the regression results. The coefficient of tobit model regression on inefficiency scores for nature log transformation of GDP per capita was 0.044 and significant at 5% level. The results suggested that higher GDP per capita associates with lower investment efficiency. We also reported the results from OLS regression whose dependent variable was not transformed by Equation 2 to check the sensitivity of our estimator. As indicated by the results, the OLS regression also identified a negative relation between GDP per capita and investment efficiency (β = −0.016, *p* < 0.05).

**Table 5:** Results of tobit model regression on inefficiency scores and OLS regression on efficiency scores.

**Variables**	**OLS model**	**Tobit model**
	**(1)**	**(2)**
GDP per capita	−0.016^*^ (0.008)	0.044^*^ (0.018)
Population	−0.001 (0.001)	0.004^*^ (0.000)
Urbanization rate	0.000 (0.000)	−0.000 (0.000)
**Hospital level (reference: primary)**		
Secondary	−0.034^***^ (0.007)	0.077^**^ (0.026)
Tertiary	−0.033^**^ (0.013)	0.072^*^ (0.036)
Un–grade	−0.015 (0.011)	0.042 (0.032)
**Whether general (reference: no)**		
Yes	0.009 (0.007)	−0.015 (0.015)
Hospital building area	0.000 (0.000)	0.000 (0.000)
Amount of investment from provincial and central government	−0.000^**^ (0.000)	0.000^***^ (0.000)
Total number of hospital beds	0.000 (0.000)	−0.000 (0.000)
Total value of equipment above 10,000 Yuan	0.000 (0.000)	−0.000 (0.000)
Number of healthcare workers	−0.000 (0.000)	0.000 (0.000)
Proportion of staff with senior professional titles	0.001 (0.002)	−0.001 (0.004)
Year dummies	Yes	Yes
*N*	318	318

*GDP is adjusted for inflation rates, and measured in 2016 RMB*.

*GDP per capita is natural log transformed in regression analysis*.

*The unit of population is 100,000; the unit of building area is square meter; the units of equipment value and amount of investment from provincial and central government are 10,000 Yuan*.

*The values of independent variables in the regression model are captured in the year of project beginning*.

*The dependent variable in the tobit model was transformed via Equation 2, while not in the OLS model*.

*Robust standard errors shown in parentheses*.

* *p < 0.05*,

** *p < 0.01*,

*** *p < 0.001*.

In terms of the other co-variables, the coefficient of population was 0.004 and significant at 5% level, which implied the similar association with efficiency like GDP per capita. Compared with primary hospitals, secondary and tertiary hospitals tend to have lower project efficiencies, with the significant coefficients reported as 0.077 and 0.072, respectively. However, un-grade hospitals had no significant difference compared with primary hospitals. The relationship between the amount of investment from provincial and central government and investment efficiency was found to be negative and even significant at 0.1% level. There were no significant differences associated with other covariates.

### Robust Test

In robust test, we modeled GDP per capita as categorical variable with 5 quantiles. Table 6 reports the results of robust test. The transformation of key independent predictor from continuous variable to categorical variable did not have a significant effect on the effect size of GDP per capita, while a higher quantile of GDP quantile was found to be associated with lower investment efficiency. However, we found a statistically significant difference merely embedded between the lowest and highest quantile of GDP per capita, which could be attributed to both the poor variation in the dependent variable and the lower power of test due to the transformation.

**Table 6:** Robust tests results.

**Variables**	**Tobit model**	
	**(1)**	**(2)**
**GDP per capita (reference: lowest quantile)**		
Secondary quantile	0.004 (0.017)	−0.003 (0.015)
Third quantile	0.020 (0.019)	−0.017 (0.016)
Fourth quantile	0.032 (0.025)	−0.027 (0.022)
Highest quantile	0.068^*^ (0.028)	−0.058^*^ (0.024)
*N*	318	318

*GDP is adjusted for inflation rates, and measured in 2016 RMB*.

*In Tobit model (1), the technical efficiency scores were transformed into inefficiency scores as Equation (2), while efficiency score was employed as outcome variable in Tobit model (2)*.

*Robust standard errors shown in parentheses*.

* *p < 0.05*.

## Discussion

In this study, we focused on hospital building construction projects as the most reflective aspect of infrastructure construction in China. Our study aims to examine whether the investment efficiency is lower where a government prioritizes equity and to explore what kind of geographical predispositions should be embedded in governmental investment plans for hospital infrastructures from the perspectives of both investment equity and efficiency. The concentration index was used to describe the distribution equity of governmental investments while tobit regression was adopted for the first research question by exploring the relationship between regional economic development and investment efficiency measured by the PCA-DEA model. Our analysis revealed slight inequity in the distribution of hospital building construction investment in Sichuan province and identified a negative relationship between county's GDP per capita and efficiency. These findings implies that the investment efficiency is higher where a government prioritizes equity since higher investment efficiency and better social equity would be achieved via predisposed resource allocation toward underdeveloped areas. Besides, our findings also illustrate that Chinese government should change the current governmental investment strategy that favors the developed regions and we provided evidence-based suggestions for regional predisposition of governmental investment toward less developed regions in terms of enhancing both equity and efficiency.

Regarding regional distribution of governmental investment in hospital building constructions, predisposition of investment allocation was found toward economically developed regions, indicating inequity in investment allocations among different regions. Despite that only two out of four sources of governmental investment, namely central and provincial governmental investments were included in our analysis, such findings were still believed to be robust. Specifically, compared with underdeveloped regions, both municipal and county governments in economically developed regions have more abundant budgets and put more emphasis on healthcare-related projects, thus tend to make more investment in hospital building construction projects. As the result, the inclusion of municipal and county governmental investments in this study, would be very much likely to produce a higher concentration index value which indicates a higher degree of inequity. Based on these considerations, our findings based on the exclusion of both municipal and county governments were considered as conservative results. At present, significant regional disparities reside in western and eastern parts of Sichuan Province in terms of the spatial accessibility to healthcare (29, 30). Under such circumstances, however, the current governmental investment predisposition toward economically developed regions continues to exacerbate the existing inequity thus further widening the gap among different regions. Therefore, optimization of governmental investment in hospital building construction is urgently needed via switching the investment predisposition toward less developed regions.

Another finding of our study was the identification of a negative relationship between the county's GDP per capita and the investment efficiency, which implied that a hospital in a county with higher GDP per capita would implement the project with lower efficiency. The underlying reasons of such negative relationship were explained from the perspectives of both demand and supply. Based on China Health and Nutrition Survey (CHNS) dataset, Xue (70) found that patients living in less developed provinces had less inclination for seeking medical treatment than patients living in economically developed provinces, which could be attributed to the lack of healthcare facilities in less developed regions. Poor spatial accessibility to healthcare typically lays tremendous hinderance for healthcare utilization (71), while there is a poorer spatial accessibility in less developed regions (30). As the consequence, it is not difficult to predict that higher efficiency would be achieved in less developed regions for hospital reconstruction, expansion or branch construction projects as these projects are desperately needed in such underdeveloped regions for meeting residents' healthcare demands. Likewise, projects related to scale upgrade or renewal of hospitals would very much likely to bring about competitive advantages for hospitals engaged in these projects in terms of attracting patients from other hospitals as the lack of large-scaled hospitals remains a critical problem in underdeveloped regions. In contrast, such competitive advantages would be largely weakened in economically developed regions due to the abundance of large-scaled hospitals in developed areas given the same investment allocations, thus leading to lower investment output and lower investment efficiency.

Despite that hospital building construction project was selected as the only focus in this study, our findings have reflected the overall situation of healthcare infrastructure construction in China to a large extent. It is noteworthy that the geographical inequality embedded in governmental investment allocations tends to escalate in the aspect of medical equipment compared with hospital building constructions. Specifically, hospitals at higher levels mainly concentrate in developed regions and typically require abundant top-tier medical facilities, thus making the predisposition of governmental investments in medical equipment promotion toward developed regions. In addition, our findings related to investment efficiency in this study could also be applied in the aspect of governmental investments in medical equipment promotions, where a list of theories about higher unsatisfied demand, higher marginal output as well as stronger competitive advantage among less developed regions as previously discussed would still be applicable.

Consistent with previous studies (27–33), our findings also provide suggestions for regional predisposition of governmental investment in healthcare infrastructure toward less developed regions. However, previous studies only assessed the locations of existing healthcare facilities from the perspective of distribution equity and failed to capture the investment efficiency across different economically developed regions. In the contrary, our study was designed to measure the investment equity and efficiency simultaneously and aimed to explore what kind of geographical predispositions should be embedded in governmental investment plans for hospital infrastructures from the perspectives of both investment equity and efficiency. Therefore, our study was endowed with the ability to provide more valid evidence-based implications for governmental investment decision-making issues.

Several limitations should be noted in this study. First, because the government funds were applied for by hospital itself, the self-selection problem may lead to the bias of the calculation of concentration index and investment efficiency. However, as most of the public hospitals in China would apply for governmental investment due to the daunting cost of hospital building constructions, such bias induced by the self-selection issue would very much likely be minimized in our study. Second, the calculation of investment efficiency could be biased in this study as we were only able to obtain governmental investment data on hospital building constructions without involving other aspects such as personnel training programs. Nevertheless, the impact of such potential confounders that we failed to involve in this study would very much likely be minimized by the first-order difference of the output variables in the DEA model with a time span of only 2 years. Further, we do not think that the potential biased efficiency scores would make a big difference to the conclusions derived from the results of regression models. In China, municipal or county governments can independently decide the amount of investment (see Text A.1 in the Supplementary Material for more details). The amount of governmental investment in hospitals is affected by numerous factors such as level of regional economic development, thought of government leaders and so on. Therefore, it would be prudent to conclude that the unmeasured governmental investments in this study could demonstrate predisposition toward economically developed regions, which indicated actual higher investment inputs based on current outputs in hospitals located in these regions. Thus, the hospitals' efficiency scores in the economically developed regions would very much likely be overestimated, leading to a dilution of the estimated negative relation between regional economic development and investment efficiency. Third, due to the lack of data related to governmental investments in other aspects, we were only able to investigate the short-term effect of hospital building construction via evaluating the investment efficiency instead of further exploring its long-term effect. Therefore, it is highly recommended that such gap be bridged in future studies once the relevant data are obtained.

## Conclusions

In the study, we revealed slight inequity in the distribution of hospital building construction investment in Sichuan province where more investment went to the economically developed regions. We also identified a negative relationship between county's GDP per capita and investment efficiency, which implied that the investment efficiency would be higher where a government prioritized equity. Combining the findings, we advocated that the governments in China should switch governmental investment predisposition in the aspect of healthcare infrastructure construction toward less developed regions from the perspectives of both investment allocation equity and efficiency. Such findings and conclusions were also expected to provide evidence-based implications for other developing countries confronted with similar governmental investment decision-making issues.

## Data Availability Statement

The information about all governmental-invested hospital building construction projects is available from the Department of Statistics of Health Commission of Sichuan Province (http://wsjkw.sc.gov.cn/scwsjkw/zsdw1/2019/3/22/9eb15f774d5549ff9a99c979fa8a0e2b.shtml), but restrictions apply to the availability of these data. The data can also be made available from the authors upon reasonable request and with permission of the Health Commission of Sichuan Province. The hospital-level data which were extracted from the hospital annual report are available from Health Commission of Sichuan Province, but restrictions apply to the availability of these data. The county-level data are publicly available, which can be obtained from the statistical yearbook of Sichuan Province. Requests to access these datasets should be directed to Jay Pan, panjie.jay@scu.edu.cn.

## Author Contributions

JP conceptualized the study. JP and TL collected the data. TL and TC developed the analysis method and implemented the data analysis. TL drafted the manuscript. JP, TL, YH, TC, and YY revised the manuscript. All authors read and approved the final manuscript.

## Funding

This work was supported by National Natural Science Foundation of China (Grant Nos. 71874116 and 72074163), Ministry of Education of China (Grant No. 18YJA790062), Chengdu Federation of Social Science Association (Grant No. ZZ05), Sichuan University (Grant Nos. 2018hhf-27 and SKSYL201811), and China Medical Board (Grant No. 17-276).

## Conflict of Interest

The authors declare that the research was conducted in the absence of any commercial or financial relationships that could be construed as a potential conflict of interest. The reviewer XL declared a shared affiliation with the authors to the handling editor at time of review.

## Publisher's Note

All claims expressed in this article are solely those of the authors and do not necessarily represent those of their affiliated organizations, or those of the publisher, the editors and the reviewers. Any product that may be evaluated in this article, or claim that may be made by its manufacturer, is not guaranteed or endorsed by the publisher.

## Footnotes

^1^Given the disparity in population density, we adjusted some variables in outcomes part by county population size. However, none of the variables in processes part was adjusted since the absolute value can directly measure the changes in the competitiveness of the hospital brought about by building construction projects, while the adjusted relative value more closely reflects the contribution of the changes of the hospital to the whole hospital market, thus the former is more in line with our investment efficiency definition.

^2^According to the leader of Health Commission of Sichuan Province and related literatures (54), there is a common situation where investment funds from municipal and county governments are apparently received by hospitals but actually not, leading to the imprecise of the data.

^3^As mentioned above, municipal or county governments can independently decide the amount of investment. Thus, the amount is affected by numerous factors such as level of regional economic development, thought of government leaders and so on, resulting in the heterogeneity and incomparability. Inclusion of the amount of investment from municipal or county government cannot reflect the regional orientation of governmental investments.

^4^According to our data, all hospitals completed the projects within two years.
