# Supplementary material for: Governmental Investments in Hospital Infrastructure Among Regions and Its Efficiency in China: An Assessment of Building Construction
Source: Front Public Health. 2021 Oct 20;9:719839. doi: 10.3389/fpubh.2021.719839 (PMC8564047; doi:10.3389/fpubh.2021.719839)
Supplement: Supplementary file 1 [file Data_Sheet_1.docx]

Supplementary Material

# Text A.1 An overview of governmental investments in hospital building construction in China

Over the past decade, China has proposed a series of investment plans for promoting nationwide hospital constructions. In Sichuan Province, for example, the annual investment (including hospital self-finance, government investment, and bank loan) reached up to 2.20 billion RMB on average, with an annual growth rate of 5.6%. In 2017 alone, the investment in hospital construction has reached 3.18 billion RMB, accounting for 9.5% of the total investment in healthcare infrastructure projects (above 5 million RMB) in Sichuan Province (1). Meanwhile, governmental investment had been serving as the main funding resource for nationwide public hospital constructions in China. In Sichuan, an annual investment of 1.25 billion RMB from both central and provincial governmental administrations was made on average, which accounted for 57.2% of the total investment.

According to the National Health Commission and the National Bureau of Statistics of China (2,3), the contents of governmental-invested hospital construction include new construction, expansion and reconstruction of office, business rooms (such as outpatients building, inpatients building, laboratory, etc.), and supportive facilities (such as canteen, parking lot, etc.).

In China, governmental investments in hospital infrastructure are typically proposed in the form of building construction projects (thus we may use building construction projects to refer to government investment in the following text) which involves two routine procedures namely project approval and funding application, as summarized in Figure A.1.

Hospitals in China can be classified into four categories according to the level of the Health Commission responsible for managing them, including: national level, provincial level, municipal level, and county level. Hospitals at different administrative levels are required to apply through different application procedures for their building constructions. For provincial, municipal, and county level hospitals, the application should be submitted to the provincial Health Commission firstly, along with the report of construction scale, amount of money, and financing source, etc. (4). After the approval, hospitals are required to submit project proposals and feasibility study reports to the provincial Development and Reform Commission, which should include the elaboration of necessity, content, site, scale, and funding of the construction project. The project would be authorized on obtaining approvals from both Health Commission and the Development and Reform Commission (5). The National Health and Health Commission is in charge of processing applications from national level hospitals. In addition to the project proposal and feasibility report, an approval document clarifying whether the construction project complies with the regional health plan should be submitted (2,6).

Funding applications for governmental investments would be typically initiated upon obtaining project approvals in the first step. There are four governmental investment categories according to the sources of government funds, namely central, provincial, municipal, and county governmental investments. However, only public hospitals which dominate China’s healthcare market by providing more than 80% outpatient and inpatient healthcare deliveries in China (7), would be eligible as recipients for governmental investments on hospital construction projects.

Regrading applications for central governmental investments, projects eligible for applications should be listed as candidates for potential healthcare investments for the upcoming three years as denoted by the central government and the rolling investment plan (8,9). This list of candidates potential for receiving central governmental healthcare investments contains a list of potential projects eligible as recipients for investments within the central government budget, which have been proposed by provincial Health Commissions along with other related departments according to planning of regional health and health resource allocation. The rolling investment plan for the next three years are extracted from the candidate list based on the regional demand. After receiving the application, the National Development and Reform Commission, the National Health Commission, and the National Administration of Traditional Chinese Medicine would determine the investment funds scale based on comprehensive consideration about the project as well as regional economic development. In principal, funding support for projects from the eastern, central, and western regions should not exceed 30%, 60%, and 80% of the total amount of project funds (10).

In terms of provincial governmental investment, all public hospitals are eligible for submitting applications to the provincial Development and Reform Commission. This authority would evaluate the applications based on multiple terms and conditions such as application justification, policy-based documentations, and whether all construction conditions required have been met in the project proposal. Following the issuing of approvals, the actual amount of provincial governmental investments would be decided based on different geographical locations, the progressive status of the construction (whether it would be a new construction, expansion or reconstruction for already existed constructions), as well as the amount of planned investment as part of the provincial governmental budget in the current calendar year (11,12).

As to applying for municipal and county investments, all applications should be submitted to municipal or county governments. Unlike all the other types of investments, currently the absence of specific terms and conditions denoting the limited percentage that recipients are allowed to receive from the total amount of investments, allows the municipal and county-level governments to invest in hospital building constructions without regulative constraints.

**Figure A.1 Summary of the project approval and funds application procedures in China**


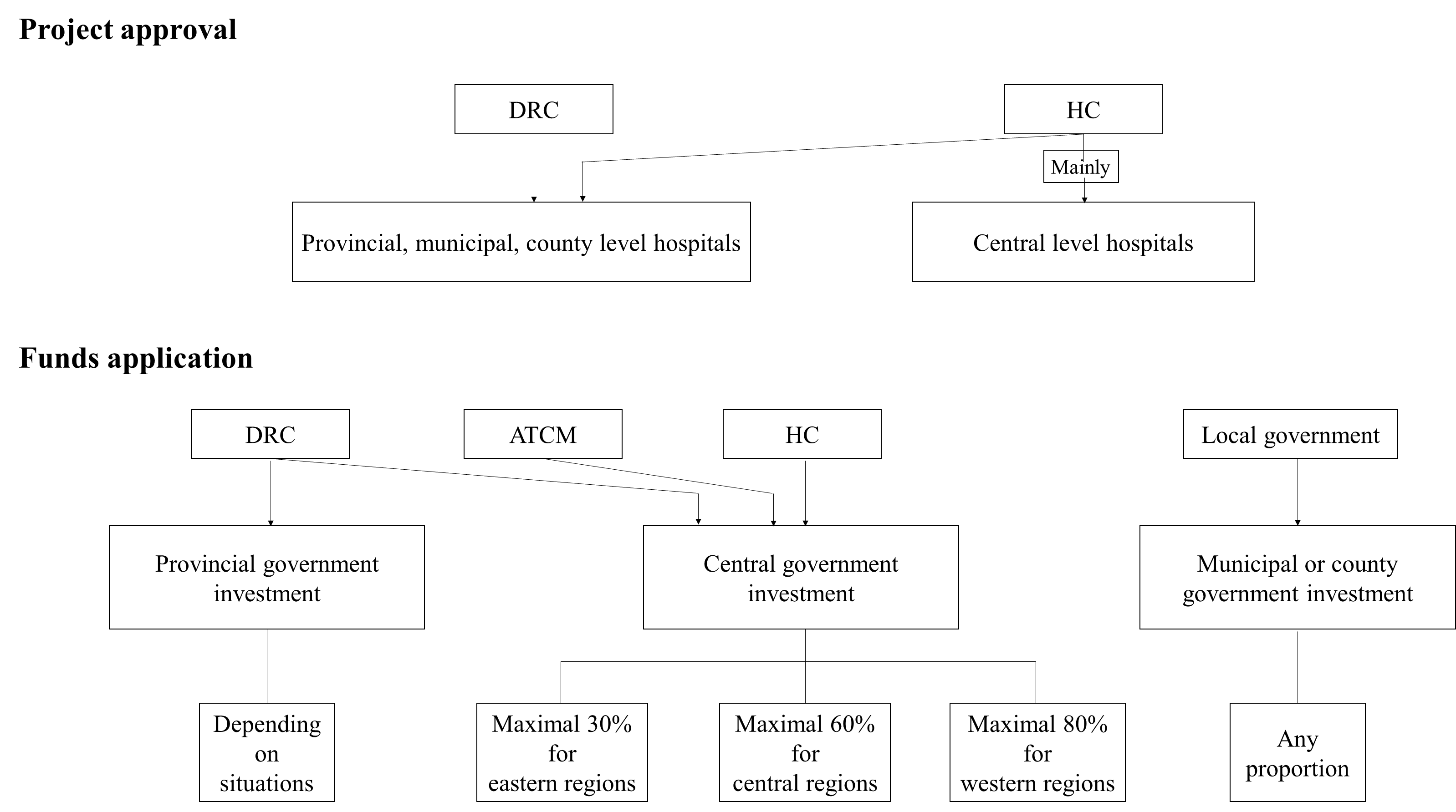


Notes: DRC=Development and Reform Commission. HC=Health Commission. ATCM= Administration of Traditional Chinese Medicine.

**Table A.1 The results of KMO test and Bartlett Spherical Detection**

| Tests | Statistics |
| --- | --- |
| KMO test | 0.952 |
| Bartlett Spherical Detection | *P*<0.001 |

**References**

1. National Bureau of Statistics of China. 2017 Statistical yearbook of the Chinese investment in fixed assests. (2018) Available at: http://www.tjcn.org/tjnj/GGG/37749.html [Accessed July 3, 2020]

2. National Health Commission of China. The management of the administration of infrastruction construction of affiliated departments of National Health Commission. (2018) Available at: http://www.nhc.gov.cn/guihuaxxs/s3585/201804/c1e287925ce341ad9893f3ea4761fad6.shtml [Accessed May 2, 2020]

3. National Bureau of Statistics of China. Investment in fixed assests. (2019) Available at: http://www.stats.gov.cn/tjsj./zbjs/201912/t20191202_1713051.html

4. Health Commission of Sichuan Province. The management of the administration of Iinfrastruction construction of affiliated departments of Health Commission of Sichuan Province (trial). (2007) Available at: http://wsjkw.sc.gov.cn/scwsjkw/ghjh/2013/1/6/cf71cdd51a7a4b079f8c94b8f80c9da9/files/7be747a2d0cf4c0fb80c33b01072a568.pdf [Accessed May 2, 2020]

5. Development and Reform Commission of Sichuan Province. The management of the administration of direct investment projects within the provincial budget of Sichuan Province. (2015) Available at: http://www.pzhsxq.gov.cn/zwgk/xwzx/bmgz/1362664.shtml [Accessed May 2, 2020]

6. Ministry of Health. The management of the administration of Iinfrastruction construction of affiliated departments of the Ministry of Health (trial). (2006) Available at: http://www.nhc.gov.cn/guihuaxxs/s3585/200804/820f036f978848aeb4e019f871031cd0.shtml [Accessed May 2, 2020]

7. National Health Commission of China. 2018 Health Statistical Yearbook of China. (2019) Available at: https://s2.51cto.com/oss/201912/05/1822362d5f7ccc8ff5d87ecdba23e64c.pdf [Accessed June 2, 2020]

8. Health Commission of Sichuan Province. The management of health construction project in Sichuan Province. (2017) Available at: http://wsjkw.sc.gov.cn/scwsjkw/ghjh/2017/5/9/aef93e05ed3e41ba9c5f721272a78db2.shtml [Accessed May 2, 2020]

9. National Development and Reform Commission. The management of investment subsidies and discount interest projects within the central government budget. (2016) Available at: http://www.gov.cn/home/2016-12/08/content_5145088.htm [Accessed May 2, 2020]

10. National Development and Reform Commission. The management of investment from the central government budget in subsidizing local medical and health construction projects. (2019) Available at: http://www.gov.cn/xinwen/2019-04/09/content_5380738.htm [Accessed May 2, 2020]

11. Development and Reform Commission of Sichuan Province. The management of the administration of investment subsidies within the provincial budget of Sichuan Province. (2015) Available at: http://www.sc.gov.cn/10462/11555/11564/2015/12/7/10361368.shtml [Accessed May 2, 2020]

12. The State Council of the Communist Party of China. Government investment rules. (2019) Available at: http://www.gov.cn/zhengce/content/2019-05/05/content_5388798.htm [Accessed May 2, 2020]
